# Effect of Therapeutic Ultrasound on Calcaneal Tendon Heating and Extensibility in Dogs

**DOI:** 10.3389/fvets.2019.00185

**Published:** 2019-06-12

**Authors:** Betzaida Acevedo, Darryl L. Millis, David Levine, Jose L. Guevara

**Affiliations:** ^1^Veterinary Orthopedic Laboratory, Department of Small Animal Clinical Sciences, University of Tennessee College of Veterinary Medicine, Knoxville, TN, United States; ^2^Department of Physical Therapy, The University of Tennessee at Chattanooga, Chattanooga, TN, United States; ^3^Department of Small Animal Clinical Sciences, University of Tennessee College of Veterinary Medicine, Knoxville, TN, United States

**Keywords:** therapeutic ultrasound, tendon temperature, tendon heating, tendon extensibility, canine calcaneal tendon

## Abstract

**Objective:** To (1) characterize the warming pattern of canine calcaneal tendons during and after four different therapeutic ultrasound (US) treatment protocols, and (2) to quantify changes in tarsal flexion immediately after therapeutic US treatment, and following return to baseline temperature.

**Design:** A prospective, crossover, experimental study.

**Animals:** Ten adult hound-type breed dogs.

**Procedure:** Therapeutic ultrasound (3.3 MHz) was applied to one calcaneal tendon of anesthetized dogs using four different settings applied in random fashion (1.5 and 1.0 W/cm^2^ continuous, and 1.5 and 1.0 W/cm^2^ pulsed US) while the temperature of the tendon was recorded by a thermistor needle. The contralateral tendon was used to compare extensibility of the treated soft tissues by measuring changes in tarsal joint flexion before, immediately after, and 5-min after continuous US treatment at 1.5 W/cm^2^ for 10 min.

**Results:** The greatest increase in tendon temperature occurred with continuous US at 1.5 W/cm^2^. Pulsed US resulted in minimal tendon heating. Most of the increase in tissue temperature occurred within the first 3 min of US application. Tarsal flexion increased significantly following US treatment; however, it returned to near baseline within 5 min after US was discontinued.

**Conclusion and Clinical Relevance:** Continuous US of the calcaneal tendon at 1.5 W/cm^2^ resulted in the greatest increase in tissue temperature while maintaining a safe range of tissue temperature increase. Tendon heating and heat dissipation were slightly different from what has been reported for muscle. Our results suggest that 3.3 MHz US applied to tendon for >3 min may not provide additional tissue temperature increase. Therapeutic US resulted in increased tarsal flexion, however the change was only transitory. Therefore, stretching exercises should be performed during and immediately after US.

## Introduction

Therapeutic ultrasound (US) is a commonly used modality for the rehabilitation of soft tissues of the musculoskeletal system. The frequency and intensity of US, the duration and duty cycle of therapy, as well as the size and cellular properties of the target tissue, interact to produce a spectrum of non-thermal (mechanical) and thermal effects. The US beam consists of high frequency (>20 kHz) ultrasound waves created by mechanical vibrations and the rapid expansion and contraction of piezoelectric crystals in the head of the US probe. The physical effects resulting from compression and rarefaction of energy are referred to as acoustic streaming and acoustic cavitation. These non-thermal phenomena have been shown to accelerate the inflammatory phase of wound healing (1), promote ion transport, increase cellular permeability (2), increase fibroblast protein synthesis (3, 4), and promote shifts in extracellular ion concentration gradients (5).

Thermal effects vary and are correlated to the magnitude of tissue warming. As the US beam penetrates into the tissues, molecules absorb energy from the waves, which increases the rate of molecular oscillation, and results in tissue warming. Previous research has demonstrated a correlation between the magnitude of temperature increase and the thermal effects produced. A mild increase of tissue temperature by 1 to 2°C, resulted in a 13% increase of the metabolic rate for each degree Celsius (6–8). A moderate increase of 3°C to 4°C has been shown to decrease pain, muscle spasm, chronic inflammation, and increase blood flow. An increase of 4°C is required to increase collagen tissue extensibility, thus improving the flexibility of the tissues, requiring less force to stretch tissues (6, 8–11).

It has also been demonstrated that the composition and morphology of individual tissues, as well as differences among species, influence the acoustic and thermal properties of the target tissue and may alter heating patterns and thermal effects. Early studies evaluated the *ex vivo* tendons of laboratory animals and discovered that heating and stretching tendons could increase tendon extensibility if applied in moderation; however, excessive heat or tension could cause irreversible damage (12–14). Additional research suggested that US energy was absorbed more efficiently in collagen dense structures, prompting these structures to heat more rapidly and to a higher temperature than adipose tissues which absorbed less energy and consequently had smaller temperature increases (15).

While a small amount of variability may be expected due to species differences, independent studies using a similar study design suggest that human muscles treated with US may increase temperature faster than canine muscles and this pattern persisted throughout the treatment period, resulting in human muscle temperatures that almost doubled compared to values reported in a canine study (16, 17). In humans, therapeutic US frequency has been widely studied concluding that US at 1.0 MHz is absorbed primarily by tissues at a depth of 3–5 cm and that a frequency of 3.3 MHz is recommended for superficial tissues at depths of l−2 cm (18). A study of dogs was performed to evaluate the effects of 3.3 MHz US of caudal thigh muscle temperature and demonstrated that significant muscle warming occurs. After US treatment for 10 min using an intensity of 1.0 W/cm^2^, muscle temperature increased 3.0°C at a depth of 1.0 cm and 2.3°C at 2.0 cm depth. Using an intensity of 1.5 W/cm^2^, the temperature increased 4.6°C at a depth of 1.0 cm, 3.6°C at 2.0 cm depth, and 2.4°C at 3.0 cm depth (17).

Distinct tissue heating patterns in different species should prompt additional investigation of tissue temperature changes with therapeutic US in dogs in order to determine appropriate protocols to provide therapeutic effects while preventing administration of sub-therapeutic or injurious treatments. Previous human studies have examined the rate of tissue warming in ligaments, the musculotendinous junction of tendons, and muscles (9, 10, 19). One study has evaluated the effect of therapeutic US on the rate and amount of muscle warming in dogs, however, to the authors' knowledge, no studies have been performed to assess the heating pattern of canine tendons treated with US, or the effect of US on the extensibility of soft tissues and its resultant effect on joint motion. The purposes of this study were to (1) characterize the warming pattern of canine calcaneal tendons during and after four different therapeutic US treatment protocols, and (2) to quantify changes in tarsal flexion immediately after therapeutic US treatment, and following return to baseline temperature.

## Materials and Methods

Ten healthy adult hound-type dogs, 4–6 years of age and weighing 17–30 Kg, were included in the study reported here. Experimental procedures were approved by the University of Tennessee Animal Care and Use Committee. A sequential two-phase repeated-measures crossover study design was performed. The first phase evaluated calcaneal tendon warming patterns with four different therapeutic US treatment protocols. The second phase assessed the effect of one protocol on the extensibility of the calcaneal tendon and related soft tissues. Before each phase, dogs were premedicated with acepromazine maleate (Boehringer Ingelheim) (0.05–0.10 mg total dose, intramuscularly) and butorphanol tartrate (Torbugesic, Fort Dodge Animal Health) (0.4 mg/kg of body weight, intramuscularly). Anesthesia was mask-induced, and dogs were intubated and maintained under general anesthesia with isoflurane in 100% oxygen. Dogs were placed in lateral recumbency on circulating warm water blankets and covered with blankets to help maintain normal core temperature.

**Figure 1:**
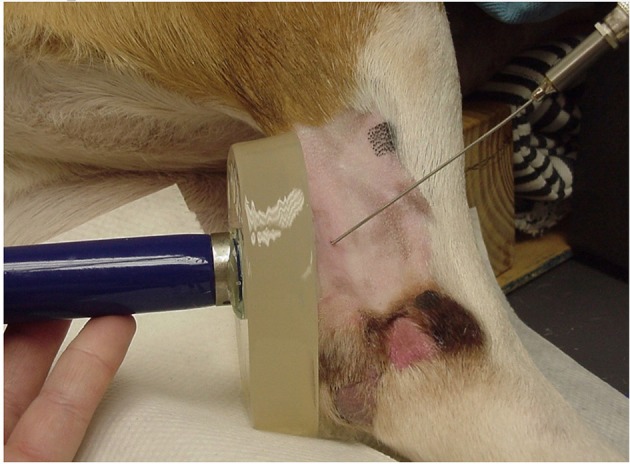
Instrumentation of a dog for data collection, showing the positions of the gel pad, US unit, and thermistor needle inserted into the calcaneal tendon (Thermistor needle aimed slightly oblique only for demonstration purposes).

The limb chosen for US treatments was randomly determined for each dog by coin toss (if heads, US was performed on the left leg first, if tails, US was performed on the right leg first). For the first phase, the hair was clipped over the caudal, medial, and lateral aspect of the calcaneal tendon, because hair has been shown to impede ultrasound transmission in dogs (20). The skin was surgically prepared with 2% chlorhexidine acetate and 70% isopropyl alcohol. The tendon and surrounding tissues were left undisturbed to allow local temperatures to stabilize. A 23 G thermistor needle (MT 23/5 Physiotemp Instruments, Clifton, NJ) was inserted into the tendon in a lateral to medial direction with the tip of the needle placed in the approximate center of the tendon. The thermistor needle was connected to a digital monitor (Dianachart Inc, Rockaway, NJ) interfaced with a computer that recorded the tissue temperature at 15-s intervals for each trial. A 1.0 cm thick 10 cm diameter gel standoff pad (AQUAFLEX, Parker Laboratories, Orange, NJ) was applied directly to the caudal aspect of the calcaneal tendon. The skin-pad and pad-US head interfaces were liberally coated with standard US transmission gel warmed to body temperature (Aquasonic 100, Parker Laboratories, Orange, NJ) (Figure 1) (21). A therapeutic US unit (Mettler Electronics, Corp., Anaheim, CA, 92805 U.S.A) was calibrated immediately prior to the study. The US was administered using a 1 cm^2^ diameter transducer head to an outlined 2 cm^2^ treatment length over the tendinous portion of the common calcaneal tendon, at a frequency of 3.3 MHz for 10 min. Dogs received four randomly ordered US treatments of continuous duty cycle at 1.0 W/cm^2^, continuous duty cycle at 1.5 W/cm^2^, pulsed 20% duty cycle at 1.0 W/cm^2^, and pulsed 20% duty cycle at 1.5 W/cm^2^. Randomization for the order of treatment in each dog was performed by drawing a treatment card from a box. After US application was initiated, tendon temperature was recorded every 15 s during the 10-min treatment period and for 10 min after US to evaluate the rate of cooling. Between treatments, temperature was recorded every 30 s for 3 min to reestablish baseline temperature of the tissue prior to application of US. After reaching baseline temperature and being certain that equilibrium was achieved, the next treatment was applied until all four treatments were completed during a single anesthetic episode.

During a separate time at least 1 week after phase one, the contralateral calcaneal tendon was prepared for the second phase. Dogs were anesthetized as previously described for the first phase and the hair was clipped over the calcaneal tendon. Goniometry was used to obtain an objective measurement of tarsal flexion. The tarsal joint was moved through a complete range of motion to determine the axis of joint rotation. The tarsal joint landmark points for goniometry were identified as previously described by Jaegger et al. (lateral aspect of the fibular head, lateral malleolus, and the proximal end of the fifth metatarsal bone) and marked with a permanent ink marker on both limbs, allowing consistent placement of the goniometer (22).

**Figure 2:**
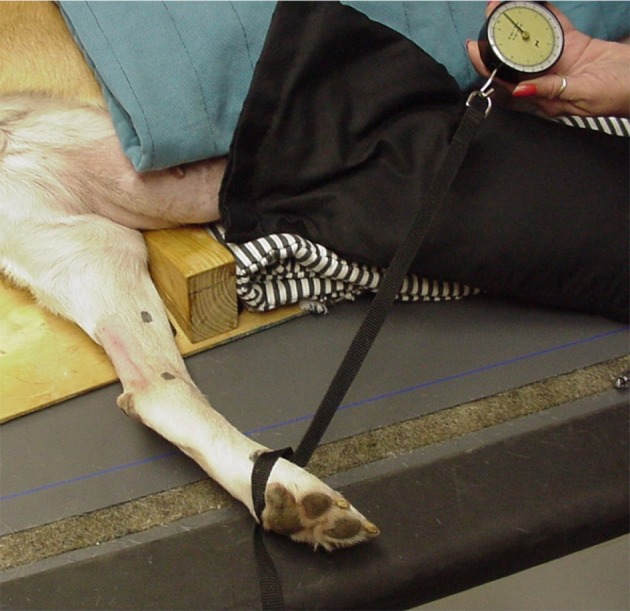
Dog with limb placed in jig to maintain stifle extension while 4.0 kg of tension (determined by use of a tension gauge) was applied to the metatarsal region for flexion of the tarsus.

Tarsal flexion with the stifle maintained in extension was used as a measure of relative tendon extensibility and was determined prior to treatment. Normally, minimal tarsal flexion occurs when the stifle is maintained in extension. If calcaneal tendon extensibility increases, tarsal flexion increases even while the stifle is extended. Dogs were placed in lateral recumbency with the limb to be measured placed in a specially designed jig to maintain the stifle joint in extension while the tarsal joint was flexed (Figure 2). A constant amount of force was used while flexing the tarsal joint by applying 4 kg of tensile force perpendicular to the metatarsal bones using a tension gauge (model PTH-AF 2, Pain Diagnostic and Treatment Corporation, Great Neck, NY). Three measurements of tarsal flexion were made, and the mean was calculated. The limb used in the first phase served as an untreated control. The untreated control was measured to ensure that any increase in tarsal flexion was due to US treatment and not because of repeated stretching. Continuous US was applied to the treated tendon at 1.5 W/cm^2^ for 10 min using the gel pad and US transmission gel because this treatment gave the greatest amount of tissue warming, while still being in the safe thermal zone. Tarsal flexion measurements were repeated as described and data recorded immediately after the US treatment on both limbs. Dogs were not manipulated or treated for the subsequent 5 min post-treatment. After the 5-min period, tarsal flexion was again measured in both the treated and untreated control limb, and mean measurements were calculated. The investigator making the measurements (DM) was blinded to treatment and control limbs.

## Statistical Analysis

Commercially available software, Statistical Package for the Social Sciences, (SPSS Inc. Version 25.0. Armonk, NY: IBM Corp.) was used to determine the average tendon temperature at each time point for each treatment (Pulsed 1.0 W/cm^2^, Pulsed 1.5 W/cm^2^, Continuous 1.0 W/cm^2^, and Continuous 1.5 W/cm^2^). No significant differences in variances and normality of data were confirmed prior to additional statistical testing. Two mixed model analyses of variance (ANOVA) with repeated measures (the first to evaluate heating during treatment, the second to evaluate cooling after treatment) were performed to compare the changes in mean tendon temperature. Significant differences between individual means were determined using a *post-hoc* least significant difference (LSD) mean separation test. Changes to the mean angle of tarsal flexion were compared with a one-way analysis of variance (ANOVA) and significant differences were evaluated *post-hoc* with a Tukey-Kramer test. Results were considered significant at *P* < 0.05.

## Results

**Figure 3:**
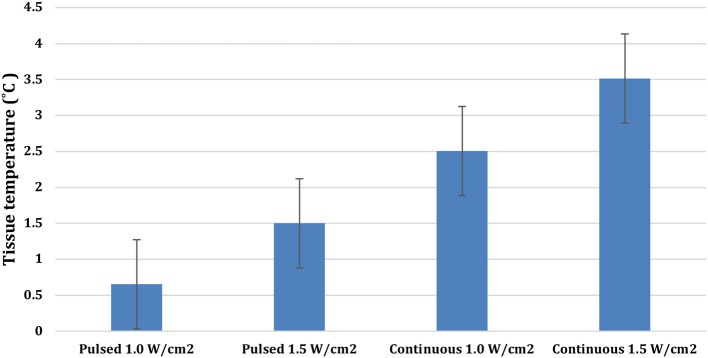
Mean temperature increase in calcaneal tendons at the end of a 10-min 3.3 MHz US treatment using the four different treatment protocols (Bars represent standard error).

**Figure 4:**
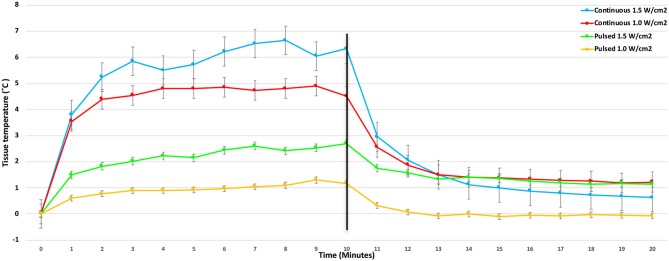
Mean change in calcaneal tendon temperature during and after the 10-min 3.3 MHz US treatment using four different treatment protocols (Bars represent standard error).

There were significant treatment, time, and treatment^*^time interactions among the US treatments applied to the tendons (*P* < 0.0001). Mean tendon temperature increases of 0.65, 1.5, 2.5, and 3.5°C were measured after 10 min of treatment with pulsed US at 1.0 W/cm^2^, pulsed US at 1.5 W/cm^2^, continuous US at 1.0 W/cm^2^, and continuous US at 1.5 W/cm^2^, respectively (Figure 3). The mean maximum tendon temperature increases were 0.9°C, 1.7°C, 3.1°C, and 4.1°C for each of the four treatments (Figure 4). At the end of the 10-min treatment time, tendons treated with continuous US at 1.5 W/cm^2^ had significantly greater temperature increase than tendons treated with pulsed US at 1.0 W/cm^2^ or 1.5 W/cm^2^ (*P* < 0.001). The increase in tendon temperature with continuous US at 1.0 W/cm^2^ was also significantly greater than tendons treated with pulsed US at 1.0 W/cm^2^ (*P* < 0.001) (Figure 4). Tendons treated with US reached maximum or near maximum temperatures in <3 min of treatment (Figure 4).

Mean temperature decreased significantly in the first minute for tendons in both of the continuous US groups (*P* < 0.001), with mean tendon temperature decreasing by more than 1°C in the 1.0 W/cm^2^ and by more than 1.8°C in the 1.5 W/cm^2^ group (Figure 4).

**Figure 5:**
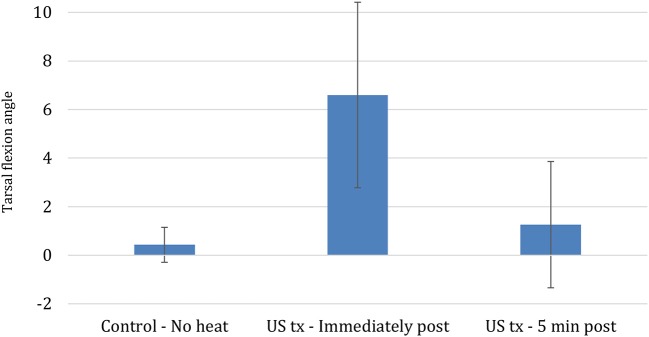
Mean change in tarsal flexion between different conditions (Bars represent standard error).

Differences in the three tarsal joint flexion measurements before calculating the mean ranged from 1 to 5 degrees, with most within 1–3 degrees. Mean tarsal flexion significantly increased by 6.6° after 10 min of 1.5 W/cm continuous US treatment, representing a 5% increase from the initial measurement (*P* < 0.001) (Figure 5). Five minutes after US was discontinued, mean tarsal flexion was not significantly greater in the treated limbs compared to the pre-US treatment values or the untreated controls (*P* < 0.773) (Figure 5). The untreated control limbs showed no significant change in mean tarsal flexion between the two measurements.

## Discussion

This is the first study describing the thermal effects of therapeutic US on canine calcaneal tendons and tendon extensibility to the authors' knowledge. The study reported here showed that treatment with continuous 3.3 MHz US at an intensity of 1.5 W/cm^2^ consistently increased canine calcaneal tendon mean temperatures *in vivo* more than 3.0°C above baseline tendon temperature. On average, tendon temperatures rose above this level <3 min after starting therapy and remained elevated for the final 7 min of treatment, yet tissue temperatures did not increase to the point that tissue damage would be expected. The mean tendon temperature increase noted with continuous US at an intensity of 1.0 W/cm^2^ was less, but still significant (Figure 3). Pulsed US treatments at intensities of 1.5 W/cm^2^ and 1.0 W/cm^2^ produced significantly less tendon temperature increase and did not increase group mean tendon temperature 3°C above baseline at any time point.

In veterinary rehabilitation, therapeutic US is commonly used to warm connective and periarticular tissues prior to performing stretching exercises. Previous studies have revealed that tissues with higher protein content, such as bone, cartilage, tendons, and ligaments, absorb a larger proportion of energy from US waves and consequently are heated more rapidly than adipose tissues, which absorb less energy and experience smaller increases in temperature (23–25). Although no study has explicitly compared differences in tissue heating between species, results from previous research suggest that while collagen heating trends may persist (collagen dense tissues absorb more energy from US waves and experience a greater temperature increase), the rate and tissue response to heating may be different in each species, and tissues within species (9, 11, 17, 18).

Tissue warming in humans has been shown to increase collagen extensibility and improve flexibility, prompting the recommendation that, to achieve optimal lengthening, tissues should be warmed prior to stretching (10, 14, 19). Previous research proposed a therapeutic temperature increase to between 40 and 45°C in people (8), however, recent studies report that many subjects do not tolerate temperatures >42°C, and heating tissues ≥45°C can result in tissue damage (11, 26, 27). Recent studies have suggested that warming tissues >3°C above baseline may result in a significantly greater range of motion, presumably as a result of the viscoelastic properties of collagen (10). In a study performed in healthy human muscle by Draper et al. thermistors were placed at depths of 2.5 and 5.0 cm in the musculotendinous junction of the triceps surae for 1.0 MHz treatment and at depths of 0.8 and 1.6 cm for 3.3 MHz treatment. The rate of temperature increased per minute at the two depths for 1.0 MHz exposure, ranged from 0.04°C at an intensity of 0.5 W/cm^2^ up to 0.38°C at an intensity of 2.0 W/cm^2^. Corresponding values for treatment with 3.3 MHz ranged from 0.3°C at an intensity of 0.5 W/cm^2^ up to 1.4°C at 2.0 W/cm^2^. The 3.3 MHz frequency heated faster at all intensities. Because these studies describe responses to US in humans, it is necessary to characterize heating patterns in the tissues of veterinary patients, since variations in thermal and acoustic tissue properties may alter the absorption and retention of US energy.

Tendons treated with continuous US at 1.5 W/cm^2^ and a frequency of 3.3 MHz exhibited significantly greater tarsal flexion immediately after treatment, increasing by 6.6° (5%) relative to flexion before heating. This finding agrees with human studies that have demonstrated an increased range of motion after US has been applied to musculotendinous junctions, however, these comparisons should be made cautiously since variations in tissue composition and architecture may produce different heating patterns (10, 11, 19, 28). The relatively small increase in tarsal flexion must also be considered in light of the sensitivity of clinical goniometry measurements. The three replicates of measurements were within 5° of each other, which is similar to the variability of 2–5° of measurements of the major joints in Labrador retrievers measured in the study by Jaegger et al. Still, the increased range was only 6.5°. The rapid loss of the increased tarsal flexion following US may have been related to a rapid decrease in tissue temperature. Based on the Phase 1 results of tendon heating with US, when tendon flexion range of motion was measured 5 min after US therapy ended, mean tendon temperature would have been only 0.56°C warmer than the baseline temperature if the temperature change in the tendon behaved similarly.

Our results regarding increased tarsal flexion with increased temperature followed by decreased flexion after cooling are consistent with previous reports that have described a “stretching window” after therapeutic US; however, this is the first confirmation of such a phenomenon occurring in canine tendons (9, 16). We believe that repeated stretching was unlikely to increase tarsal flexion because the limb of the control non-heated tendon did not exhibit changes in tarsal flexion with repeated measurements. A similar study using human Achilles tendons also found that ultrasound and stretch increased mean dorsiflexion range of motion in all sessions significantly more than stretch alone (10).

Our study confirmed that canine calcaneal tendons could be heated with therapeutic US to improve tendon extensibility; however, the magnitude and rate of tendon heating differed from the expected pattern. Studies performed in humans by Draper et al. and Chan et al. supported this idea and have demonstrated that under similar treatment settings (1.0 W/cm^2^ at 3 MHz administered over an area equivalent to 2 effective radiating areas (ERA- 4.5 cm^2^) in human muscle, at a depth of 0.8 cm, temperature increased 5.8°C over a 10-min. US treatment with an average of 0.6°C/minute, compared with the human patellar tendon which reported a temperature increase of 8.3°C over a 4-min US treatment with an average rate of 2.1°C/minute (9, 16). This rate of temperature increase in tendon was 3.45 times faster than in muscle.

Using similar therapeutic US settings (1.0 W/cm^2^ and a frequency of 3.3 MHz), Levine et al. demonstrated that temperature increase at the end of 10-min treatment time in canine muscle at a depth of 1 cm was 3.0, 2.3°C at a depth of 2.0 cm, and at a depth of 3 cm was 1.6°C. In this study, canine calcaneal tendons (~1 cm thick) treated with the same settings resulted in a mean temperature increase of 2.5°C at the end of treatment. In the first minute of treatment, mean calcaneal tendon temperature increased by 2.0°C when US was applied at an intensity of 1.0 W/cm^2^, and by 2.1°C when US was applied at an intensity of 1.5 W/cm^2^. The different heating patterns and the maximum magnitude of temperature change were likely associated with the energy produced by the US probe at the two intensities. These values reveal that canine muscle appears to have a relatively steady increase in tissue temperature during US treatment, while canine calcaneal tendon temperature in this study increased significantly faster within the first 2 min, and then stayed almost constant for ~8 min of treatment time.

A recent, similar study published by Montgomery et al. evaluated temperature change of equine superficial digital flexor tendons using continuous 3.3 MHz US treatment at 1.0 or 1.5 W/cm^2^ intensities for 10 min. Temperatures increased 3.5 and 5.2°C for the 1.0 and 1.5 W/cm^2^ intensities, respectively. Furthermore, temperature rose 2°C after 3 min using 1.0 W/cm^2^, and within 2 min using 1.5 W/cm^2^. Compared to our study, canine calcaneal tendon temperature increased 2°C in the first minute using the two different treatment settings, which is slightly faster.

Rehabilitation requires a gradual return to use with special attention to gradual stretching of tendons to encourage return to normal function over time. The thermal effects of US may have relevance in the treatment of a variety of musculoskeletal conditions in the dog. Clinical application of tendon heating and extensibility could be considered for the treatment of conditions that can cause tightening or contraction of tendons which is not uncommon following an injury or after prolonged immobilization periods required for a tendon injury to heal. However, caution is recommended in applying US heating and stretching of healing tendon injuries to avoid laxity or breakdown of tendon repairs. When applying therapeutic US to tissue it is difficult to predict the thermal effects in an individual patient because tissue heating is influenced by a number of factors including tissue density and origin, anatomic location, properties of surrounding structures, blood flow, stage in the course of healing, and body composition. Additional variability may also have been associated with the systemic and peripheral changes secondary to general anesthesia, which is not used in the clinical application of US. The purpose of general anesthesia in this study was simply to allow the thermistors to be placed and maintained without undue discomfort to the dogs.

The authors believe that the external environment should also be considered as a source of variability since tissues dissipate heat more rapidly in an environment with colder ambient temperatures. Also, environmental heat loss may play a significant role in the heating and cooling patterns exhibited in canine calcaneal tendons because these structures are less vascular and minimally covered by other tissues in the distal third of the pelvic limb, potentially making them more susceptible to changes in environmental temperatures. To minimize the influence of these variables, dogs were maintained in a room with constant temperature and were maintained on circulating warm water blankets and covered with blankets to help maintain core body temperature. Another possible source of tissue heating with US is that the thermistor needle could have been heated with US, and the heat generated by this process may have been transferred to the tissues in addition to US treatment of the tendon (29). However, the power used on cadaver tissues in that study ranged from 40 to 600 W/cm^2^, while we used 1 and 1.5 W/cm^2^. The heating artifact at 40 W/cm^2^ was relatively small, and this power of US likely would have caused severe tissue damage. In addition, the thermistors used in our study are designed to minimize thermal conduction.

The authors acknowledge a number of limitations in the present study that warrant further investigation. Warming patterns were only evaluated in the common calcaneal tendon in a small population of morphologically similar healthy dogs. Other tendons may have different tissue properties including insulation by fat and muscle which may alter absorption and retention of US energy. Additionally, the administration of anesthetic agents may alter thermoregulatory controls and it is unclear if tendons experience secondary effects as well. Future studies examining the thermal effect of US on a variety of tendons and different body types may provide a more complete characterization of the heating patterns and extensibility in canine tendons.

In conclusion, a 10-min session of continuous US applied with an intensity of 1.5 W/cm^2^ at a frequency of 3.3 MHz was capable of increasing mean canine calcaneal temperatures more than 3.0°C. Calcaneal tendons heated in this fashion with application of a controlled stretch force immediately after treatment exhibited significantly greater tarsal flexion angles possibly due to increased extensibility of the calcaneal tendon following treatment. However, the increase was temporary and after a 5-min rest period, there was no significant difference between mean tarsal flexion angles in the treated and control groups. The temporary nature of the increase in tarsal flexion is consistent with an increase in tendon extensibility secondary to the changes in viscoelastic properties in tissues when they are heated.

## Ethics Statement

Experimental procedures were approved by the University of Tennessee Animal Care and Use Committee. These were laboratory animals, and therefore there was no owner consent.

## Author Contributions

We certify that all authors meet the qualifications for authorship as listed below: (1) substantial contributions to the conception or design of the work or the acquisition, analysis, or interpretation of data for the work; (2) drafting the work or revising it critically for important intellectual content; (3) final approval of the version to be published; (4) agreement to be accountable for all aspects of the work in ensuring that questions related to the accuracy or integrity of any part of the work are appropriately investigated and resolved.

### Conflict of Interest Statement

The authors declare that the research was conducted in the absence of any commercial or financial relationships that could be construed as a potential conflict of interest.
